# Irreversible aggregation of alternating tetra-block-like amphiphile in water

**DOI:** 10.1371/journal.pone.0202816

**Published:** 2018-08-27

**Authors:** Shota Konno, Taisuke Banno, Hideaki Takagi, Satoshi Honda, Taro Toyota

**Affiliations:** 1 Department of Basic Science, The University of Tokyo, Komaba, Meguro-ku, Tokyo, Japan; 2 Department of Applied Chemistry, Faculty of Science and Technology, Keio University, Hiyoshi, Kohoku-ku, Yokohama, Japan; 3 Photon Factory, Institute of Materials Structure Science, High Energy Accelerator Research Organization, Oho, Tsukuba, Ibaraki, Japan; University of Michigan, UNITED STATES

## Abstract

As a frontier topic of soft condensed matter physics, irreversible aggregation has drawn attention for a better understanding of the complex behavior of biomaterials. In this study, we have described the synthesis of an artificial amphiphilic molecule, an alternating tetra-block-like amphiphile, which was able to diversify its aggregate structure in water. The aggregated state of its aqueous dispersion was obtained by slow evaporation of the organic solvent at room temperature, and it collapsed irreversibly at ~ 50°C. By using a cryo-transmission electron microscope and a differential scanning calorimeter, it was revealed that two types of molecular nanostructures were formed and developed into submicro- and micrometer-sized fibrils in the aggregated material.

## Introduction

Generally, hydrogels formed by artificially synthesized polymers and surfactants reversibly harden or soften in response to changes in temperature. Some water-containing aggregates of biomolecules that constitute everyday foods become hard even when low concentrations of biomolecules are mixed with water. These aggregates often respond irreversibly to temperature. For instance, yoghurt is comprised of casein micelles, which have diameters in the range of several decanometers to submicrometer,[1] and exhibits separation into water and precipitates when heated up. This phenomenon is termed as irreversible aggregation.[2] While several attempts have been made to elucidate the mechanism of irreversible aggregation with model systems using colloidal fine particles,[3–5] as far as we know, experimental reports using organic molecules have been almost limited to dispersions of biomaterials (such as proteins, polysaccharides, blood platelets etc) which sometimes have difficulty of material identification.

**Fig 1:**
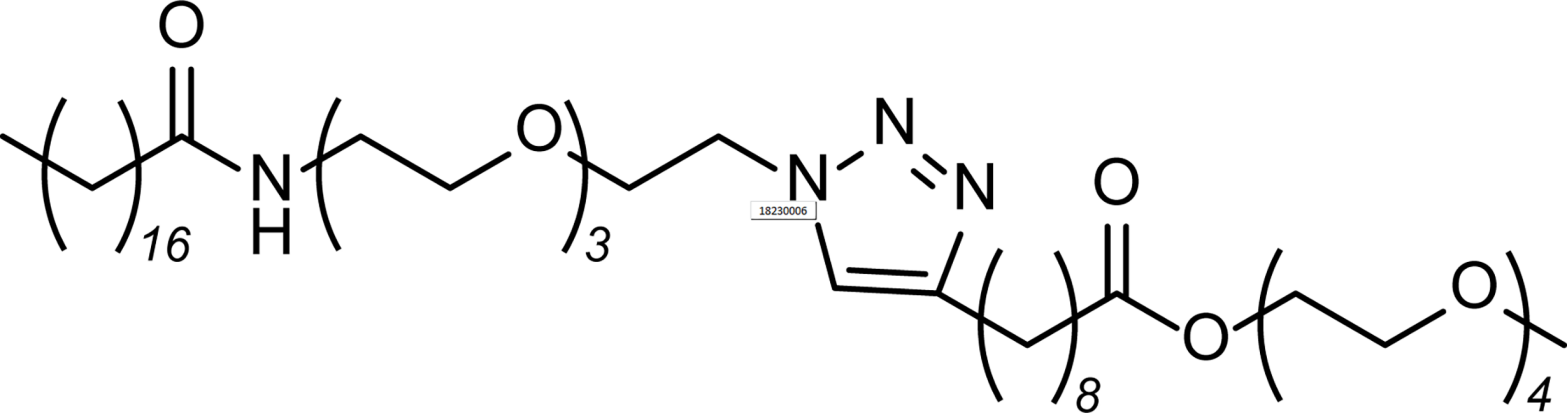
Scheme of alternating tetra-block-like amphiphile ATBA.

The theoretical description of irreversible aggregation is based on the diversified structure of aggregated particles beyond their length scale.[6,7] Recent development on cryo-transmission electron microscopy[8,9] and methodologies on magnetic nuclear resonance[10–14] as well as X-ray/neutron scattering measurement[15,16] contribute to explore the mechanical properties of polymer and aggregated particles. Pavišic et al experimentally demonstrated the reversible and irreversible aggregation of a human cytokine having an overall size of several nanometers and evaluated their crossover changing upon the addition of saccarides.[17] Besides of such biomolecules, irreversible aggregation of graphene was also observed in the reduction process of graphene oxide.[18] Moreover, Corezzi et al. have numerically investigated the irreversible aggregation mechanism using percolating clusters[19] composed of two types of molecules that can interact with each other via their coarse-grained affinity sites.[20] Considering that the biomaterials affording irreversible aggregation are usually hard to be identified in terms of purity and that the impurities affect the aggregation structures and kinetics[21–23], an amphiphile with exactly-determined but precisely imbalanced chain lengths was newly synthesized in this study. This alternating tetra-block-like amphiphile **ATBA** is composed of two hydrophilic modules of oligo(ethylene oxides) and imbalanced hydrophobic alkyl chains, where the length of one of the two hydrophobic alkyl chains is precisely twice as long as that of the other (Fig 1). Since one of the two alkyl chains is twice as longer as the other, and linked to the oligo(ethylene oxide) chains with a different bond (ester bond) than the amide linkage, the alignment of **ATBA** owing to the molecular interaction in water is expected to vary with the conformations of its alkyl chain. In order to avoid monotonous micelle formation, referring to the chemical structures of Tween 20 and Tween 80 which are well-known micelle-forming surfactants, the total length of the oligo(ethylene oxide) chains of **ATBA** is less than the half of that of those surfactants. We expected that this imbalance would lead to irreversible aggregation in association with diversification of the molecular alignment patterns.

## Materials and methods

### Reagents

11-Azido-3,6,9-trioxaundecan-1-amine (>93.0%), 10-undecynoic acid (>98.0%), tetraethylene glycol monomethyl ether (>98.0%), and 1-(3-dimethylaminopropyl)-3-ethylcarbodiimide (>98.0%) were purchased from TCI (Japan). Stearic acid (>95.0%), 4-dimethylaminopyridine (>99.0%), and CuCl (>95.0%) were procured from Wako Chemicals (Japan). *N*,*N'*-dicyclohexylcarbodiimide (>98.0%) was purchased from Kanto Chemical (Japan). All solvents and reagents were used as received. ^1^H- and ^13^C-NMR spectra were recorded on an AV-500 spectrometer (Bruker, USA) operating at 500 MHz and 125 MHz respectively. CDCl_3_ was used as the solvent. ^1^H-NMR spectra were referenced to tetramethylsilane (TMS) and ^13^C-NMR spectra were referenced to the deuterated solvent CDCl_3_. High resolution electrospray ionization mass spectrometry (HR-ESI-MS) was performed by using a JMS-T100LC spectrometer (JEOL, Japan).

### Synthesis

#### Synthesis of (N-11-azido-3,6,9-trioxaundecylamine)octadecanoate (1)

A CH_2_Cl_2_ (3 mL) solution of 4-dimethylaminopyridine (244 mg, 2.00 mmol) was added to a mixture of 11-azido-3,6,9-trioxaundecan-1-amine (1.31 g, 6.00 mmol), stearic acid (1.43 g, 5.03 mmol), and *N*,*N'*-dicyclohexylcarbodiimide (1.03 g, 4.99 mmol) in CH_2_Cl_2_ (25 mL). The reaction mixture was stirred at room temperature under Ar atmosphere for 7 h. Subsequently, the mixture was filtered and the filtrate was evaporated. The residue was purified by silica gel column chromatography using *n*-hexane/EtOAc (1/2) as the first eluent and CHCl_3_/MeOH (4/1) as the second eluent. The second eluent was concentrated to dryness to obtain **1** (1.39 g, 57%) as a white powder.

(*N*-11-azido-3,6,9-trioxaundecylamine)octadecanoate (**1**): Yield 57%. ^1^H-NMR (500 MHz, CDCl_3_): δ (ppm) 5.99 (1H, s,–N*H*–), 3.70–3.65 (8H, m,–OC*H*_*2*_C*H*_*2*_–), 3.64–3.62 (2H, m, N_3_C*H*_*2*_–), 3.56 (2H, t, *J* = 5.08 Hz,–NHCH_2_C*H*_*2*_–), 3.46 (2H, quartet, *J* = 5.22 Hz,–NHC*H*_*2*_–), 3.39 (2H, t, *J* = 5.08 Hz, N_3_CH_2_C*H*_*2*_–), 2.17 (2H, t, *J* = 7.65 Hz,–C*H*_*2*_CO–), 1.62 (2H, quint, *J* = 7.44 Hz,–C*H*_*2*_CH_2_CO–), 1.33 (28H, br,–CH_2_–), 0.90 (3H, t, *J* = 6.98 Hz,–CH_3_). ^13^C-NMR (125 MHz, CDCl_3_): δ(ppm) 171.36, 70.70, 70.60, 70.56, 70.22, 70.05, 69.94, 50.64, 39.08, 36.75, 31.89, 29.67, 29.67, 29.67, 29.67, 29.67, 29.63, 29.63, 29.60, 29.48, 29.36, 29.33, 29.31, 25.72, 22.65, 14.09.

#### Synthesis of 2,5,8,11-tetraoxatridecyl-10'-undecynoate (2)

A CH_2_Cl_2_ (3 mL) solution of 4-dimethylaminopyridine (244 mg, 2.00 mmol) was added to a mixture of 10-undecynoic acid (0.911 g, 5.00 mmol), tetraethylene glycol monomethyl ether (1.25 g, 6.00 mmol), and 1-(3-dimethylaminopropyl)-3-ethylcarbodiimide (776 mg, 5.00 mmol) in CH_2_Cl_2_ (30 mL). The reaction was carried out with stirring at room temperature under Ar atmosphere. After 6 h, the reaction mixture was diluted by the addition of CHCl_3_ (100 mL) and the solution was washed sequentially with 3 M HCl (3 × 150 mL), 3 M NaOH (150 mL), and brine (150 mL). The organic layer was concentrated and purified by silica gel column chromatography using *n*-hexane/EtOAc (1/2) to obtain **2** as a colorless oil (1.26 g, 68%).

2,5,8,11-tetraoxatridecyl-10'-undecynoate (**2**): Yield 68%. ^1^H-NMR (500 MHz, CDCl_3_): δ (ppm) 4.22 (2H, t, *J* = 4.85 Hz,–COOC*H*_*2*_–), 3.70 (2H, t, *J* = 4.88 Hz,–COOCH_2_C*H*_*2*_–), 3.68–3.63 (10H, m,–OC*H*_*2*_C*H*_*2*_–), 3.56–3.54 (2H, m, CH_3_OC*H*_*2–*_), 3.38 (3H, s, -OC*H*_*3*_), 2.33 (2H, t, *J* = 7.58 Hz,–C*H*_*2*_CO–), 2.17 (2H, td, *J* = 3.56 Hz, 2.62 Hz,–CC*H*_*2*_–), 1.94 (1H, t, *J* = 2.65 Hz,–CC*H*), 1.62 (2H, quint, *J* = 7.28 Hz,–COCH_2_C*H*_*2*_–), 1.53 (2H, quint, *J* = 7.29 Hz,–CCCH_2_C*H*_*2*_–), 1.43–1.35 (2H, quint,–COCH_2_CH_2_C*H*_*2*_–), 1.35–1.25 (6H, br,–C*H*_*2*_–). ^13^C-NMR (125 MHz, CDCl_3_) δ(ppm): 173.73, 86.64, 71.89, 70.57, 70.57, 70.54, 70.51, 70.48, 69.16, 68.07, 63.32, 58.97, 34.12, 29.04, 28.99, 28.83, 28.60, 28.37, 24.80, 18.31.

#### Copper(I)-catalyzed azide alkyne cycloaddition (CuAAC) reaction of 1 and 2

Weighed amounts of **1** (1.35 g, 2.79 mmol), **2** (1.06 g, 2.84 mmol), and CuCl (0.285 g, 2.88 mmol) were dispersed in water (29 mL) and stirred at room temperature for 24 h. Subsequently, the mixture was diluted with CHCl_3_ (120 mL) and the solution was washed with 5.3% (w/v) citric acid (3 × 150 mL). The organic layer was concentrated to dryness to obtain a white, waxy solid. The crude product was purified by silica gel column chromatography using *n*-hexane/EtOAc/acetone (1/4/1) as the first eluent and CHCl_3_/MeOH (4/1) as the second eluent to obtain **ATBA** as a white powder (1.88 g, 79%).

Product of CuAAC reaction (**ATBA**): Yield 79%. ^1^H-NMR (500 MHz, CDCl_3_): δ (ppm) 7.41 (1H, s,–C = C*H*–), 6.04 (1H, br,–N*H*–), 4.50 (2H, t, *J* = 5.23 Hz,–NNNC*H*_*2*_–), 4.22 (2H, t, *J* = 4.88 Hz,–COOC*H*_*2*_–), 3.88 (2H, t, *J* = 5.23 Hz,–NNNCH_2_C*H*_*2*_–), 3.70 (2H, t, *J* = 4.88 Hz, −COOCH_2_C*H*_*2*_–), 3.67–3.58 (18H, m,–OC*H*_*2*_C*H*_*2*_–), 3.56–3.54 (2H, m, CH_3_OC*H*_*2*_–), 3.54 (2H, t, *J* = 4.27 Hz,–NHCH_2_C*H*_*2*_–), 3.45 (2H, q, *J* = 5.28 Hz,–NHC*H*_*2*_–), 3.38 (3H, s,–OC*H*_*3*_), 2.69 (2H, t, *J* = 7.73Hz, = CC*H*_*2*_–), 2.32 (2H, t, *J* = 7.68 Hz,–OCOC*H*_*2*_–), 2.17 (2H, t, *J* = 7.65 Hz,–NHCOC*H*_*2*_–), 1.70 (2H, m, = CCH_2_C*H*_*2*_–), 1.62 (4H, m,–NHCOCH_2_C*H*_*2*_–,–OCOCH_2_C*H*_*2*_–), 1.45–1.25 (36H, br,–C*H*_*2*_–), 0.88 (3H, t, *J* = 6.98 Hz,–C*H*_*3*_). ^13^C-NMR (125 MHz, CDCl_3_): δ (ppm) 172.82, 172.65, 147.24, 121.08, 71.22, 69.89, 69.89, 69.86, 69.84, 69.81, 69.79, 69.77, 69.77, 69.47, 69.16, 68.89, 68.43, 62.43, 58.19, 49.32, 38.40, 35.80, 33.39, 31.21, 28.99, 28.99, 28.99, 28.99, 28.99, 28.99, 28.97, 28.95, 28.95, 28.84, 28.78, 28.73, 28.65, 28.65, 28.47, 28.47, 28.47, 28.35, 25.09, 24.94, 24.15, 21.97, 13.44. HRMS [M+Na^+^] calcd. for C_46_H_88_N_4_O_10_ 879.6398, found 879.6366.

### Differential scanning calorimetry (DSC)

**Fig 2:**
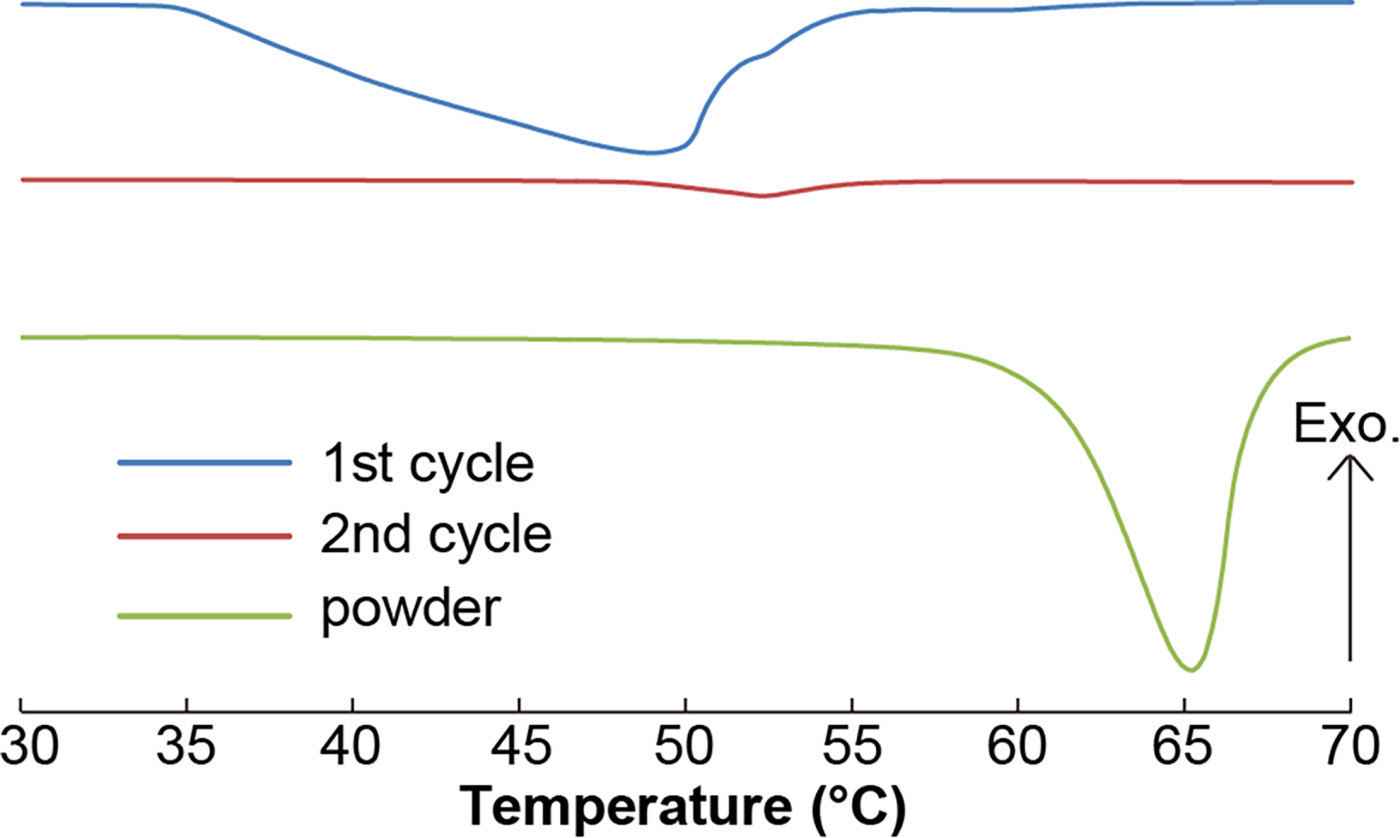
DSC profiles of ATBA_G_ (first cycle: Blue line, second cycle: Red line) and ATBA in powder state (green line).

An aqueous solution of **ATBA** (31 mg) was prepared in THF/water (5/1 v/v, 6 mL). The aggregated state of the dispersion (**ATBA**_**G**_) was formed by slow evaporation of THF for two days from the mixture at ambient temperature (ca. 20°C). Before the DSC measurement, we added water to **ATBA**_**G**_ for preparation of the sample with a 40 μL aluminum pan (final weight of the sample; 25 mg, **ATBA** conc.; 3wt%). The measurements were then performed in the temperature range of 0–70°C using a DSC 3 (Mettler Toledo, USA) instrument; the heating rate was 5°C min^–1^. At the beginning of each measurement, the samples were held isothermally for 5 min at 0°C. The result of the 2nd heating cycle is shown in Fig 2 to eliminate the effect of the thermal history. For reference, the powder of ATBA (9.2 mg) in the same procedure was also measured.

### Cryo-transmission electron microscopy (TEM)

**ATBA**_**G**_ was formed by the slow evaporation of THF from the mixture of **ATBA** (31 mg) and THF/water (3/1 v/v, 4 mL) at ambient temperature (ca. 20°C) and diluted with water (1 mL) before observation by cryo-TEM. This diluted **ATBA**_**G**_ (2 μL) was stained with 2% (w/v) uranyl acetate (2 μL) and placed on a microgrid with holes of 1 μm in diameter. The excess suspension was blotted with a piece of filter paper. After blotting, the grid was quickly frozen in liquid ethane by using a Leica EM GP (Leica) system and transferred into liquid nitrogen until required. The grid in the Cryo-Transfer Holder G914 (Gatan, USA) was observed by using a JEM-2100F (JEOL, Japan) instrument operating at 200 kV.

### Optical microscopy

**ATBA**_**G**_ was observed using the IX71 phase contrast microscope (Olympus, Japan) equipped with CCD camera DP72 (Olympus, Japan). **ATBA**_**G**_ was prepared as mentioned above and, for the observation sample, it was sandwiched (thickness ~ 50 μm) with two cover glasses (Matsunami, No.1, Japan) and a double-sided tape (400P50, Kyodo giken chemical, Japan). We also observed **ATBA**_**G**_ (thickness ~ 0.3 mm, Frame Seal Chamber, MJ Research Inc., Waltham) sandwiched with two cover glasses under a polarized optical microscope (Olympus BX50-P, lens; UPLANFI10X, N.A.; 0.30).

### Wide-angle X-ray scattering (WAXS)

WAXS measurement was performed with a D8 ADVANCE diffractometer (Bruker) using a Lynx Eye detector. The X-ray wavelength used for this measurement was 1.542 Å (CuKα radiation). The instrument was operated at a voltage of 40 kV and a current of 40 mA. The samples were scanned at a rate of 10 s per step with a step width of 0.03° in the range of 2*θ* = 3–14°. For sample preparation, an aqueous solution of **ATBA** (31 mg) was prepared in THF/water (3/1, 4 mL). **ATBA**_**G**_ was formed by slow evaporation of THF from the mixture at ambient temperature (ca. 20°C). Furthermore, the powder of **ATBA** and the white precipitate formed in **ATBA**_**G**_ by the thermal cycle, which involved heating above 50°C and cooling to room temperature, were also evaluated.

### Synchrotron radiation small-angle X-ray scattering (SAXS)

SAXS data collection was carried out at the beamline BL-10C at the Photon Factory (Tsukuba, Japan).[24] The X-ray wavelength used for this measurement was 1.5 Å. The sample-to-detector distance was 1066 mm and 3025 mm; and the calibration was performed using silver behenate as a standard sample. These experimental set-ups provided a detectable Q-range of order 0.1–6.35 and 0.015–1.09 nm^−1^, respectively. In BL-10C, the X-ray beam was generated by a bending magnet. The beam was monochromatized by a double crystal monochromator and focused by a bent-cylindrical mirror. The focused beam was cut by the second slit (beam-size definition slit), which was located just behind the monochromator. The parasitic noise scattering induced by the collimation slits was guarded by the scatterless slit (Xenocs) installed just before the sample position on the diffractometer. The beam size was V 0.3 mm × H 0.6 mm at the scatterless slit. The beamstop size was V 5 mm × H 6 mm. The scattering of **ATBA**_**G**_ was recorded with a PILATUS3 2M detector (DECTRIS), which had pixel size of 172 μm and the detection areas of V 179.4 mm × H 168.7 mm.[25] Each two-dimensional scattering image was circularly averaged to convert the one-dimensional scattering intensity data; data processing was performed with an SAngler.[26] The scattering intensity was corrected for background scattering and sample absorption. The magnitude of the scattering vector (*q*) is given by: *q* = 4πsin(*θ*/2)/*λ*, where *λ* is the wavelength of the X-ray and *θ* is the scattering angle. For sample preparation, an aqueous solution of **ATBA** (31 mg) was dissolved in THF/water (3/1, 4 mL). **ATBA**_**G**_ was formed by slow evaporation of THF from the mixture at ambient temperature (ca. 20°C). Temperature-dependent SAXS analysis of **ATBA**_**G**_ was performed at a heating rate of 5°C min^–1^ in the temperature range of 25–60°C. The exposure time was 10 s. Just before accumulating the data, an X-ray was not irradiated to the sample to avoid the X-ray damage.

### Rheological analysis

An aqueous solution of **ATBA** (31 mg) was prepared in THF/water (3/1 v/v, 4 mL) and slow evaporation of THF from the mixture at ambient temperature (ca. 20°C) afforded **ATBA**_**G**_. As reference sample, we also prepared an aqueous suspension of **ATBA** (**ATBA**_**S**_) by mixing **ATBA** (62 mg) in water (2 mL) for 3 h and allowing the mixture to stand for 22 h at room temperature (ca. 20°C). Dynamic mechanical analysis (DMA) was performed with the HAAKE MARS III (Thermo Scientific, USA) instrument. The sample cell was a circular glass-dish with a diameter of 32 mm. The circular flat-plate probe for DMA had the diameter of 20 mm and a glasspaper (Sankyo Rikagaku, PSpaper 180) was pasted on the probe surface. The sample press-in depths of **ATBA**_**G**_ and **ATBA**_**S**_ were 2 mm and 0.5 mm respectively. Frequency sweep test was carried out in the range of 0.6–62 rad/s with constant strain for **ATBA**_**G**_ (0.2%) and **ATBA**_**S**_ (0.5%) respectively. From 0.6 to 1.0 rad s^−1^, we observed a slight increase in *G’* and slight decrease in *G”* in both of **ATBA**_**G**_ and **ATBA**_**S**_. We then omitted this region as a drift of artifact. The strains of 1.0–62 rad s^−1^ were in the linear viscoelastic region of each sample. The stress sweep measurement was performed in the range of 0.1–100% at constant frequency (1 rad s^−1^).

## Results

### Aggregation behavior of ATBA in water

The synthesis of **ATBA** was carried out via the CuAAC reaction between the azide- and ethynyl-functionalized precursors, which were synthesized according to modified literature procedures[27,28] (S1 and S2 Figs). When we dispersed **ATBA** powder in water in the concentration range of 4–16 wt%, a highly viscous state with **ATBA** concentration of over 10 wt% appeared at ambient temperature (~20°C). When this viscous dispersion was heated to over 55°C, water started to separate and the precipitation occurred eventually. On the other hand, we also prepared aqueous solution of **ATBA** by dissolving powdered **ATBA** into THF/water mixtures (1/1–3/1, v/v) in range of 0.1–2.5 wt% and slowly evaporated THF from the solution at ambient temperature (ca. 20°C). It was found that the solution became highly viscous after 48 h of THF evaporation. The removal of THF was confirmed by ^1^H NMR analysis (S3 Fig) and the final concentration of **ATBA** was determined to be 5 ± 2 wt% by weight analyses. Thus, the **ATBA** dispersion exhibited the aggregated state even at concentrations less than 10 wt%. This aggregated dispersion formed by the method of THF evaporation, **ATBA**_**G**_, visually collapsed at 50°C and a white precipitate was formed after cooling to ambient temperature. ^1^H NMR analysis of the precipitate of heated **ATBA**_**G**_ revealed that the heating process did not decompose **ATBA** at the molecular level (data not shown). The WAXS profile of the precipitate of heated **ATBA**_**G**_ appeared to be similar to that of **ATBA** powder (S4 Fig), indicating that the former was not an equilibrium state; and instead, the coexistence of **ATBA** solid and water was probably more stable.

### Thermal property of ATBA_G_

The thermal properties of **ATBA**_**G**_ were examined by DSC. A broad endothermic peak was observed at 35–55°C in the first heating cycle of the DSC curve of **ATBA**_**G**_; this peak almost disappeared in the second heating cycle (Fig 2, blue line). The intensity of the endothermic peak declined noticeably compared to that of powder **ATBA** (Fig 2, green line); the broadness of the peak indicates the existence of multiple molecular alignment patterns within **ATBA**_**G**_. Further, the calculated enthalpy (Δ*H*) associated with the endothermic peak in the first heating cycle was 124 J g^–1^. Given that the Δ*H* of powder state **ATBA** was 112 J g^–1^ and the concentration of **ATBA** in the sample of **ATBA**_**G**_ was 3 wt%, the assumed Δ*H* for melting of **ATBA** as an individual component of the mixture was ca. 3 J g^–1^. Hence, the remaining portion of Δ*H* of 121 J g^–1^ was plausibly derived from nanostructures formed within **ATBA**_**G**_. Comparison of the first and second cycles of the DSC curves (Fig 2, red line) also showed that the nanostructures collapsed in the first heating cycle.

### Microscopy observation of ATBA_G_ in nanometer and micrometer scale

**Fig 3:**
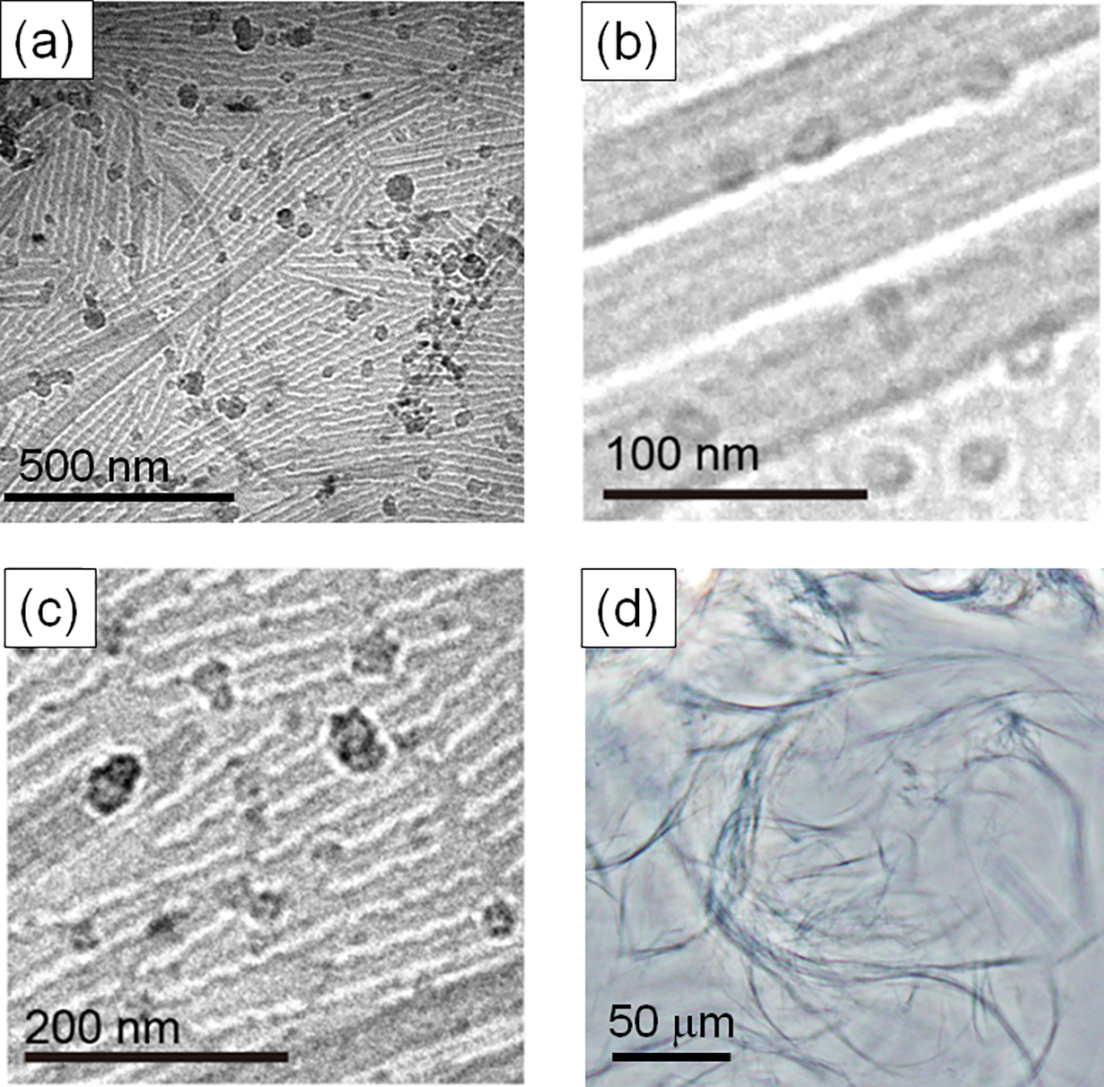
(a–c) Cryo-TEM images and (d) optical microscope image of **ATBA**_**G**_.

Cryo-TEM was used to directly observe the formed structures within **ATBA**_**G**_. The cryo-TEM images of **ATBA**_**G**_ showed two completely different nanostructures (Fig 3A). The first type of nanostructure was thread-like and had an average width of 37–39 nm with stripe patterns parallel to the direction of the tube (Fig 3B). The interval of these patterns was estimated to be 6–12 nm from the image, which corresponded to the extended chain length of **ATBA** (ca 7 nm). The thread-like nanostructures were locally entangled in a bent manner in the TEM images (S5 Fig). The second type of nanostructures had periodical patterns (Fig 3C). Relating to this, Han and coworkers have previously reported the presence of mixed morphologies of nanocylinders and stripes with the self-assembly of their block copolymers, and also found that the solvent evaporation time of the block copolymer/solvent mixture affected the ratio of the nanocylinder and stripe morphologies.[29] Hence, the present mixed morphologies observed in Fig 3A–3C were likely due to the locally varying concentration of **ATBA** and water within **ATBA**_**G**_. Moreover, the optical microscope image of **ATBA**_**G**_ showed curved fibrils having diameters of 2–3 μm (Fig 3D). The birefringence of **ATBA**_**G**_ appeared under a polarized optical microscope and the fingerprint texture in the polarized optical microscope image demonstrated similarity with those of lyotropic liquid crystallines (S6 Fig).[30,31] Taken together, nanometer- to micrometer-size structures were successfully formed by the aggregation of **ATBA**.

### WAXS and SAXS measurement of ATBA_G_

**Fig 4:**
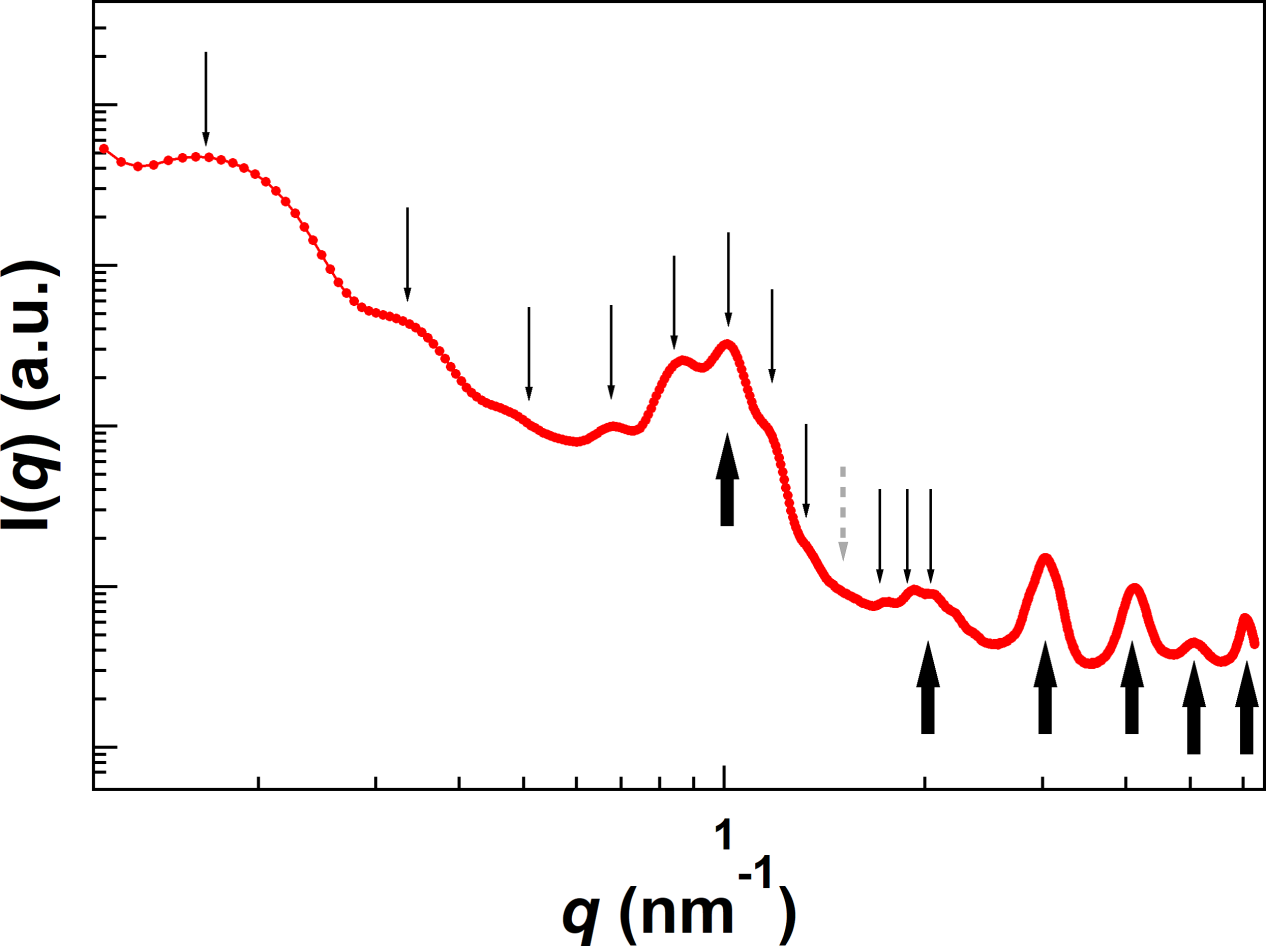
SAXS profile of ATBA_G_. Thin and thick arrows represent the scattering peak positions of the lamella structure having *d* = 36 nm and 6.2 nm, respectively. Gray dotted arrow means the missing 9th peak position.

Besides of cryo-TEM, WAXS and SAXS measurement and analysis are powerful to explore the nano/denanometer-sized structure of **ATBA**_**G**_. The WAXS profiles shown in S4 Fig indicated that the solid state of **ATBA** is lamellar structure with a distance of ca. 6 nm (3.04, 4.12, 5.09, and 6.10 nm^–1^). The first and second peaks of WAXS profiles are missing due to the lower q-range limit of WAXS. We found that the synchrotron radiation SAXS profiles demonstrates two distinct lamellar structures in **ATBA**_**G**_ (Fig 4).[32] The scattering peaks were observed at the relative peak position of 1: 2.02 : 2.96 : 4.08 : 5.04 : 6.05 : 7.1 : 8.06 : 10.1 : 11.2 : 12.1 and 1 : 1.96 : 3 : 4.08 : 5.01 : 5.98, as indicated by thin and thick arrows in Fig 4, respectively. Both of these scattering peak ratios were seen as multiple integers, indicating the formation of two types of lamellar structures. The long periods of the lamellar structure were calculated by *d* = 2π/q*, where q* refers to the first order peak position. The value of *d* was determined to be 36 nm (thin arrows) and 6.2 nm (thick arrows). Thus, it was found that the lamellar structures of 36 nm and 6.2 nm periods coexisted in the aggregation of **ATBA**_**G**_. Although we could not determine the origin of the width of the thread and the inner structure of each thread in terms of these lamella structures, this structural analysis certainly indicates the presence of characteristic mesoscopic structures in the aggregated material **ATBA**_**G**_.

S7 Fig shows the temperature-dependent synchrotron radiation SAXS analysis of **ATBA**_**G**_. The structural collapse was not observed in the decanometer region (0.1–1.5 nm^–1^) at 35–50°C. The peaks in the decanometer region disappeared at 50°C. This roughly corresponded to the aforementioned collapse of the nanostructures within **ATBA**_**G**_ that was also observed by DSC analysis (Fig 2). The second cycle of the temperature-dependent SAXS analysis revealed partial irreversible collapse of the formed nanostructures upon heating. At 25°C in the second cycle, the scattering peaks observed in the first cycle disappeared, and broad scattering peaks appeared in the range of 0.15–0.25 nm^–1^ and 0.5–1.3 nm^–1^.

### Rheological analysis of ATBA_G_

**Fig 5:**
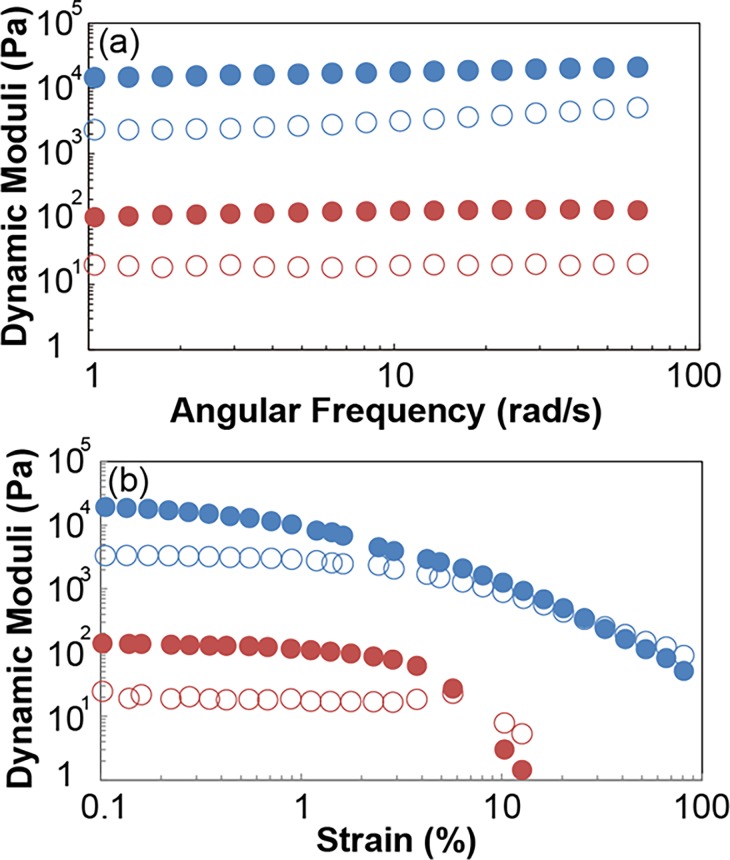
(a) Frequency-dependence of *G*’ (filled circle) and *G*” (blanked circle) for **ATBA**_**G**_ (blue) and **ATBA**_**S**_ (red). (b) Strain-dependence of *G*’ (filled square) and *G*” (blanked square) for **ATBA**_**G**_ (blue) and **ATBA**_**S**_ (red).

Although discrete nanometer- and micrometer-size structures were observed within **ATBA**_**G**_, it is difficult to define the relationship between these constituents for the present system. Nevertheless, pioneering efforts have revealed that structural diversity and hierarchy affect the mechanical properties of various systems.[33–36] Inspired by this, dynamic mechanical analysis (DMA) of **ATBA**_**G**_ was performed. The storage modulus (*G*’) and loss modulus (*G*”) of **ATBA**_**G**_ were measured at constant low strain and determined to be 17,700 Pa and 3,150 Pa for 10 rad·s^–1^, respectively, whereas the *G*’ and *G*” values for the suspension of **ATBA** with the similar concentration prepared by simply mixing with water (see Supporting Information) were 133 Pa and 19.4 Pa, respectively (Fig 5A). These results indicated that both *G*’ and *G*” were almost independent of the frequency in the range of 1–62 rad·s^–1^ (where *G*’ was consistently greater than *G*”), confirming that the rheological behavior of the analyzed samples was more similar to that of a solid than to that of a liquid. The strain-dependence of *G*’ and *G*” for each sample was also examined under constant frequency to estimate the yield stress. As shown in Fig 5B, with increasing strain, there was a decline in *G*’ for both samples and *G*” became larger than *G*’. It has been reported that the yield stress of a material can be estimated from the applied stress at the crossover point.[37] In this experiment, the yield stress of the viscous mixture was estimated to be 113 Pa by using the crossover method, whereas that of the suspension was 0.87 Pa. Finally, from the results of the frequency sweep and strain sweep experiments, it was evident that there were clear differences in *G*’, *G*” yield strain and yield stress for **ATBA**_**G**_ and the suspension of **ATBA**. The mechanical properties of the aggregated state of **ATBA**_**G**_ mixture were enhanced and found to be comparable to that of previously reported hydrogels. For example, the *G*’ value for 3% chitin nanofiber gel that was covalently bonded to a polysaccharide was reported to be 13 kPa, and the *G*’ for **ATBA**_**G**_ had a comparable value.[38] Thus, the mechanical properties of **ATBA**_**G**_ were likely influenced by the formation of the diversified aggregation of **ATBA** molecules.

## Discussion

Recent theoretical research in colloidal science has focused on frameworks describing irreversible aggregation. Most of these are based on the aggregation of spherical colloidal particles having attractive interaction.[2–6] In the case of attractive colloids[39], the uniform suspension state is stable only when the temperature is high or the attractive force is weak. Once the temperature decreases or the attraction force between colloids increases, the system becomes unstable against phase separation into colloids-rich and medium-rich regions. During this phase separation, the dynamics gets highly sluggish, resulting in the irreversible formation of colloidal gel. A similar mechanism may play a role in the present system; when **ATBA** was dispersed into the THF/water mixture, the uniform solution state was stable. However, after THF was excluded, the system became unstable against phase separation as in the case of the colloidal gel, resulting in the irreversible formation of **ATBA**_**G**._ Since **ATBA** molecules formed thread-like nanostructures and nanometer-scaled periodical patterns in **ATBA**_**G**_, the direct description of the mechanism of formation of **ATBA**_**G**_ on a molecular and intermolecular level is simply over-speculation. However, since the thread-like and periodical nanostructures in **ATBA**_**G**_ were able to entangle and form an aggregate, such as the highly viscous state of multilamellar tube dispersion,[40] the surface of these nano/decanometer-sized structures in **ATBA**_**G**_ was closed to each other. This may induce local hydrophobic interaction as weak attractive interactions between these nano/decanometer-sized structures in **ATBA**_**G**_ because the short alkyl chain and the triazole ring are sandwiched with the hydrophilic ethylene oxide chains. Upon heating, the **ATBA** molecules melted and the ethylene oxide chains of **ATBA** molecule lost the surrounding water molecules,[41,42] Consequently, the water molecules escaped from the nano/decanometer-sized structure, causing the collapse of the aggregated state.

## Conclusions

In this work, we achieved mesoscopic structural diversity ranging from the nanometer to micrometer scale in the aggregated material **ATBA**_**G**_. DSC analysis suggested that nanostructures derived from multiple molecular alignments were formed in **ATBA**_**G**_ because of the presence of water, which collapsed upon heating above 50°C. This conclusion was supported by temperature-dependent SAXS analysis and the direct observation of these nanostructures using cryo-TEM. Furthermore, optical microscopy revealed the presence of micrometer-scale fibrils in **ATBA**_**G**_, which confirmed its diversified structures. The mechanical properties of **ATBA**_**G**_ were dramatically enhanced compared to that of **ATBA**_**S**_ based on DMA, and this difference could be attributed to the nanostructural diversity. Although the multiple potential molecular alignments of **ATBA** are veiled in terms of the contribution of the asymmetric structure to the complex nanostructure and mechanism of formation of **ATBA**_**G**_ in the dissipation system generated by THF evaporation, the current findings provide a novel experimental model for irreversible aggregation.

## Supporting information

S1 Fig ^1^H NMR spectra of (a) **1**, (b) **2**, and (c) **ATBA**.
(TIF)

S2 Fig ^13^C NMR spectra of (a) **1**, (b) **2**, and (c) **ATBA**.
(TIF)

S3 Fig ^1^H NMR spectra of (a) **ATBA**_**G**_ and (b) THF.
(TIF)

S4 Fig WAXS profiles of powder-state ATBA (blue line), ATBA_G_ (red line), and heated ATBA_G_ (greenline). For sample preparation, an aqueous solution of **ATBA** (31 mg) was prepared in THF/water (3/1, 4 mL). **ATBA**_**G**_ was formed by the slow evaporation of THF from the mixture at ambient temperature (ca. 20°C). Heated **ATBA**_**G**_ was the white precipitate formed from **ATBA**_**G**_ in the thermal cycle that involved heating it above 50°C and then cooling to room temperature. In the WAXS profile of the powder-state **ATBA**, the peaks attributed to the crystals in the powder appeared at 3.0, 4.1, 5.1, and 6.1 nm^-1^, whereas they disappeared in the profile of **ATBA**_**G**_. Thus, the structure of the aggregated material on a scale below 3 nm was likely to be different from that of the powder. In the WAXS profile of the heated **ATBA**_**G**_, the peaks appeared at 3.1, 4.3, 5.2, and 6.2 nm^-1^ and the position of these peaks were similar to that of powder-state **ATBA**. These results suggest that the structure of the white precipitate resembled the crystal structure in powder-state **ATBA**.
(TIF)

S5 Fig Flexible feature of thread-like nanostructure of ATBA_G_ in Fig 3 of the main text. (TIF)

S6 Fig Polarized optical microscopy image of ATBA_G_. (TIF)

S7 Fig SAXS profiles of ATBA_G_ with increasing temperature: ((a) first cycle, (b) second cycle).
(TIF)
