# Correction: Irreversible aggregation of alternating tetra-block-like amphiphile in water

**DOI:** 10.1371/journal.pone.0205140

**Published:** 2018-09-28

**Authors:** The PLOS ONE staff

Fig 1 is incorrect. The text box with numbers “18230006” should not appear in the figure. The publisher apologizes for the error. Please see the corrected Fig 1 here.

**Fig 1:**
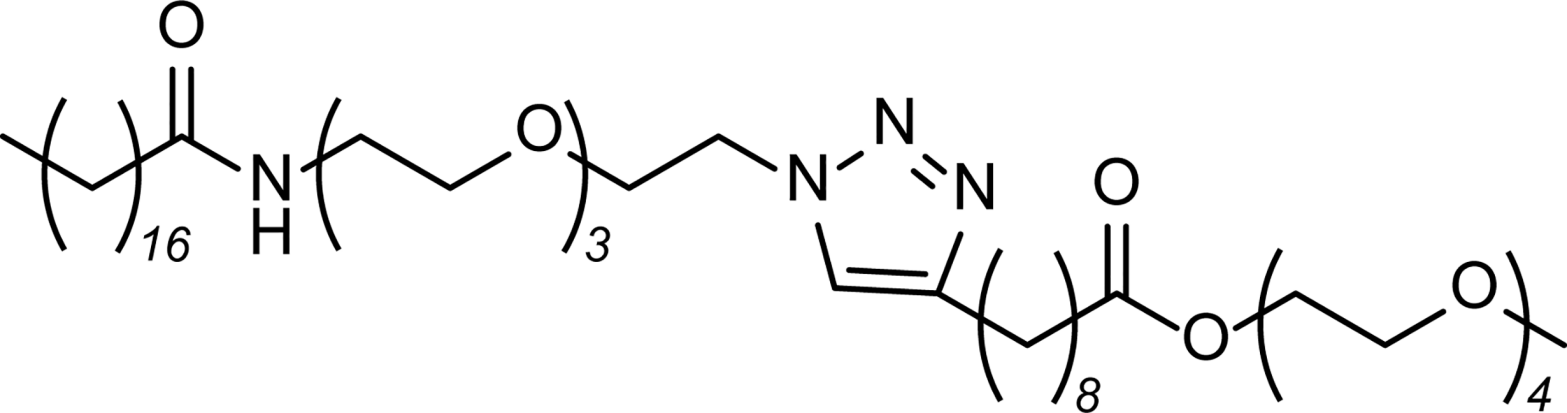
Scheme of alternating tetra-block-like amphiphile ATBA.
