# Supplementary figures and images for: Irreversible aggregation of alternating tetra-block-like amphiphile in water
Source: PLoS One. 2018 Aug 27;13(8):e0202816. doi: 10.1371/journal.pone.0202816 (PMC6110477; doi:10.1371/journal.pone.0202816)

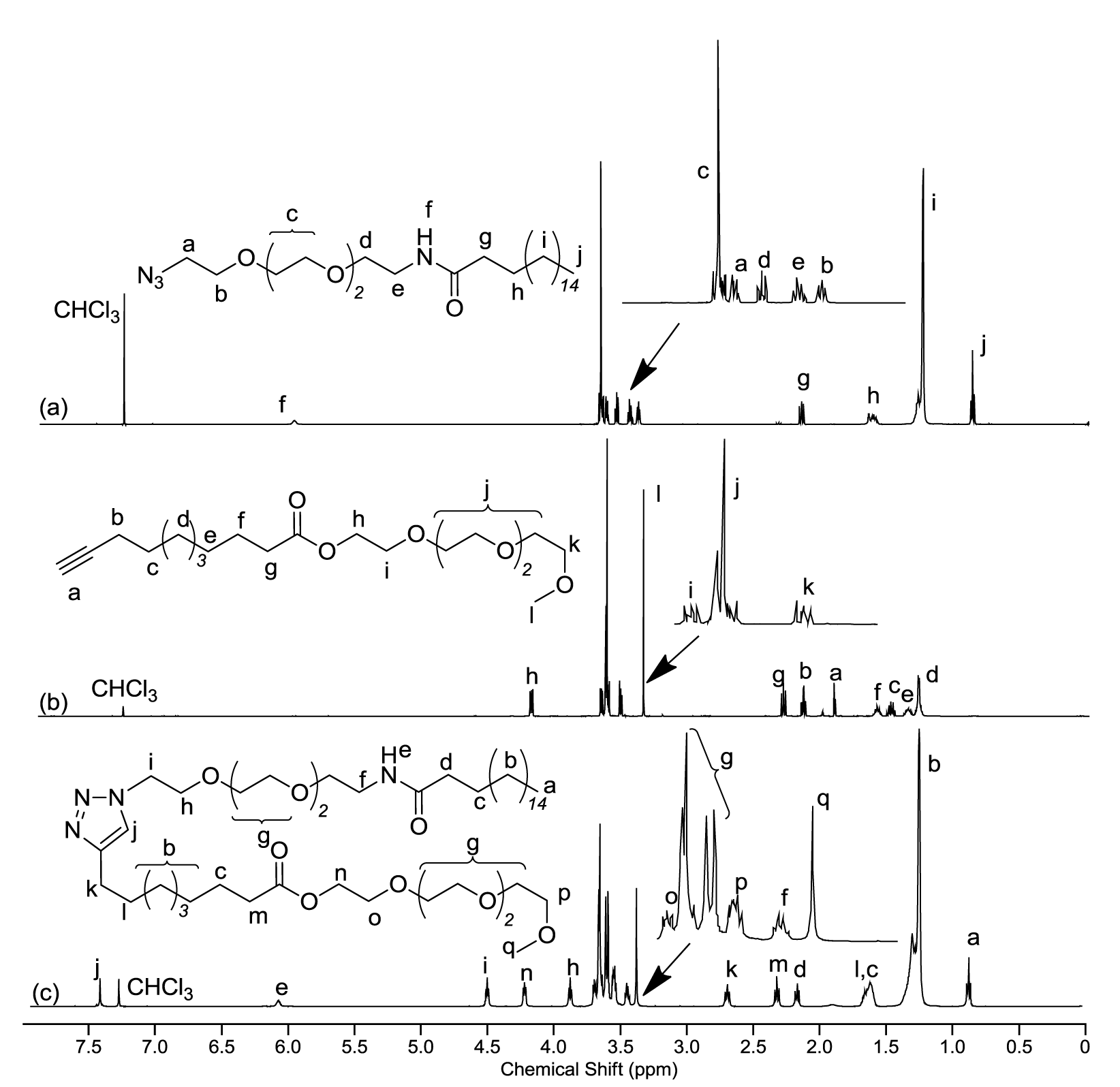

Supplement: S1 Fig — 1H NMR spectra of (a) 1, (b) 2, and (c) ATBA. (TIF) [file pone.0202816.s001.tif]

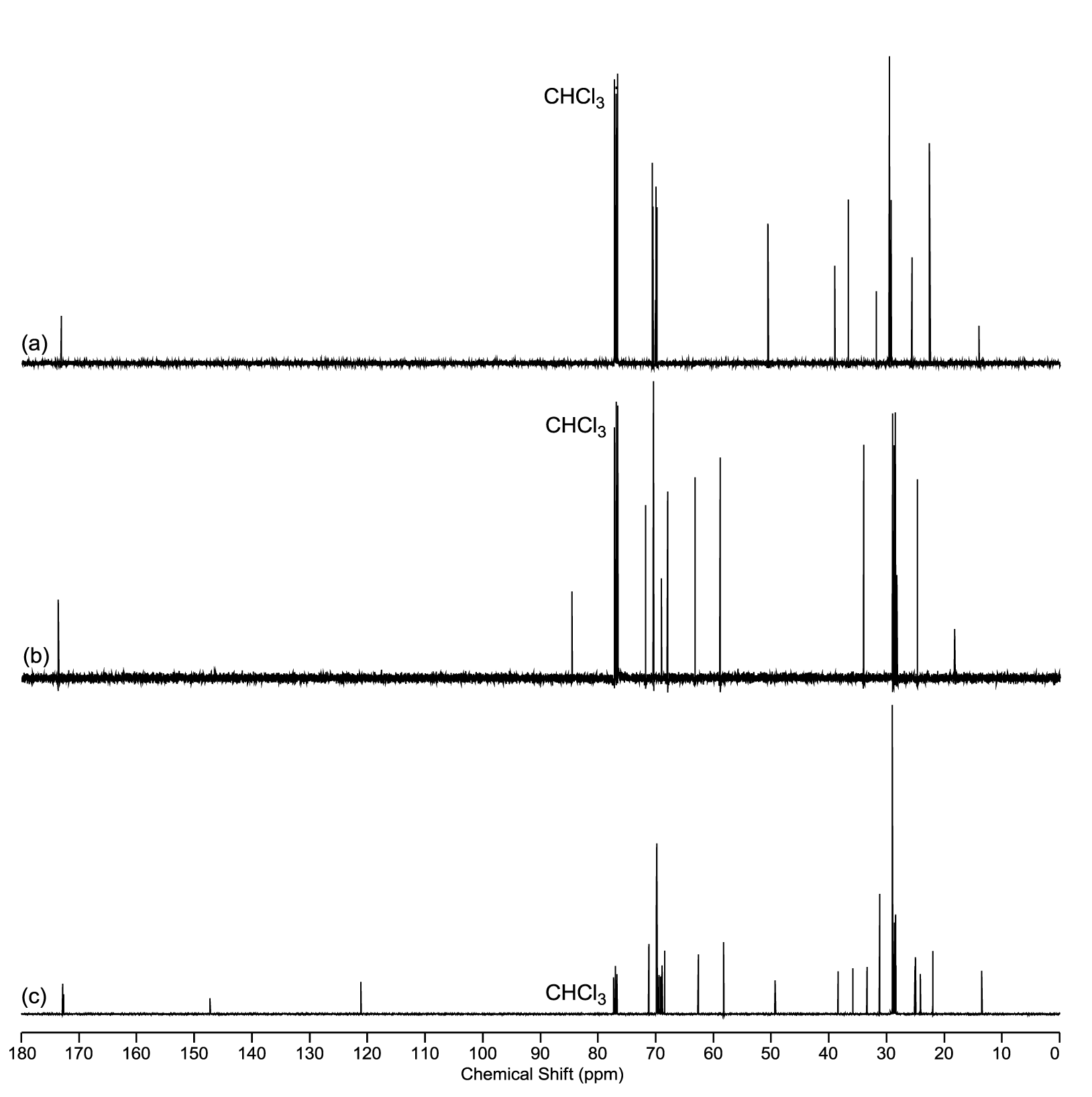

Supplement: S2 Fig — 13C NMR spectra of (a) 1, (b) 2, and (c) ATBA. (TIF) [file pone.0202816.s002.tif]

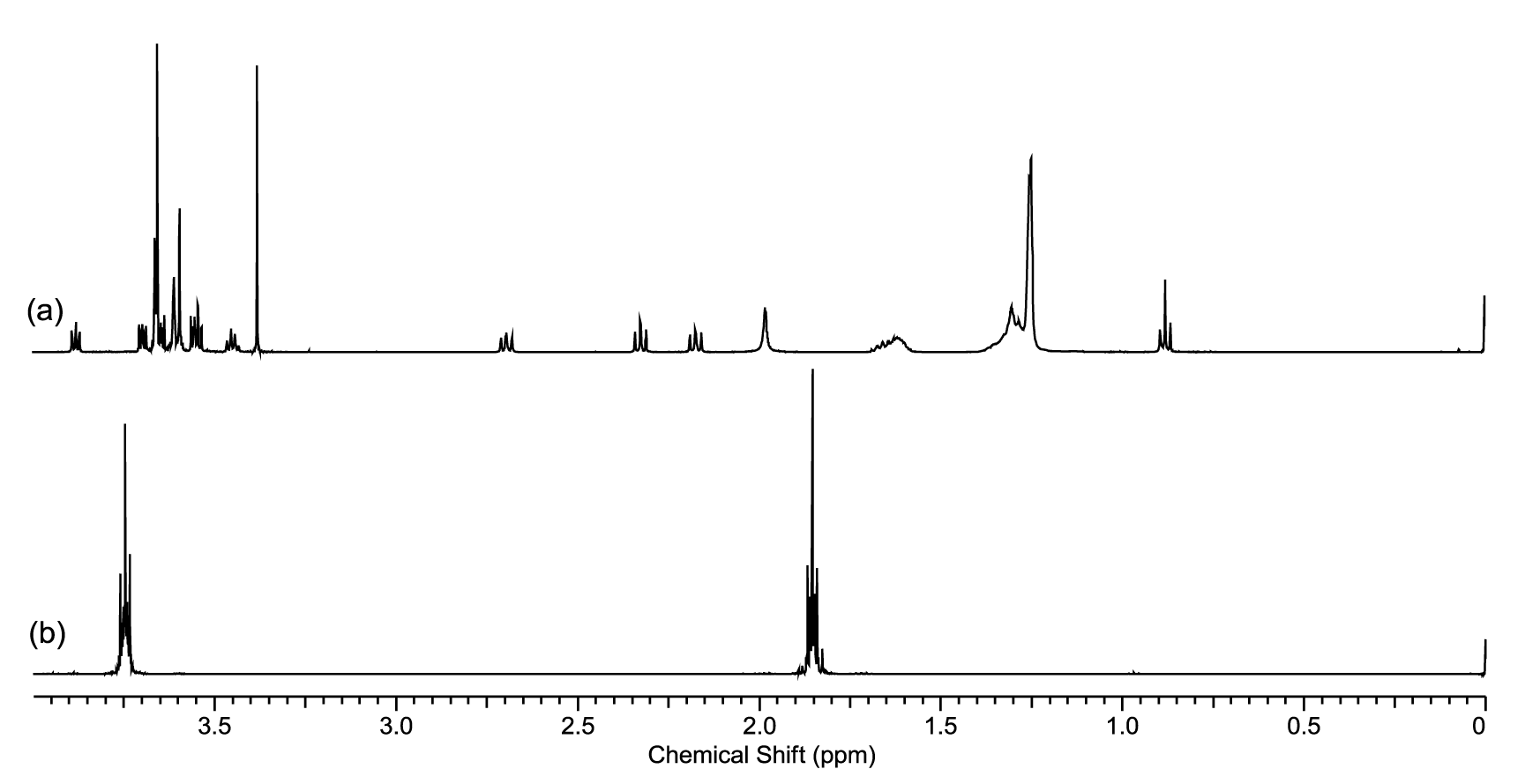

Supplement: S3 Fig — 1H NMR spectra of (a) ATBAG and (b) THF. (TIF) [file pone.0202816.s003.tif]

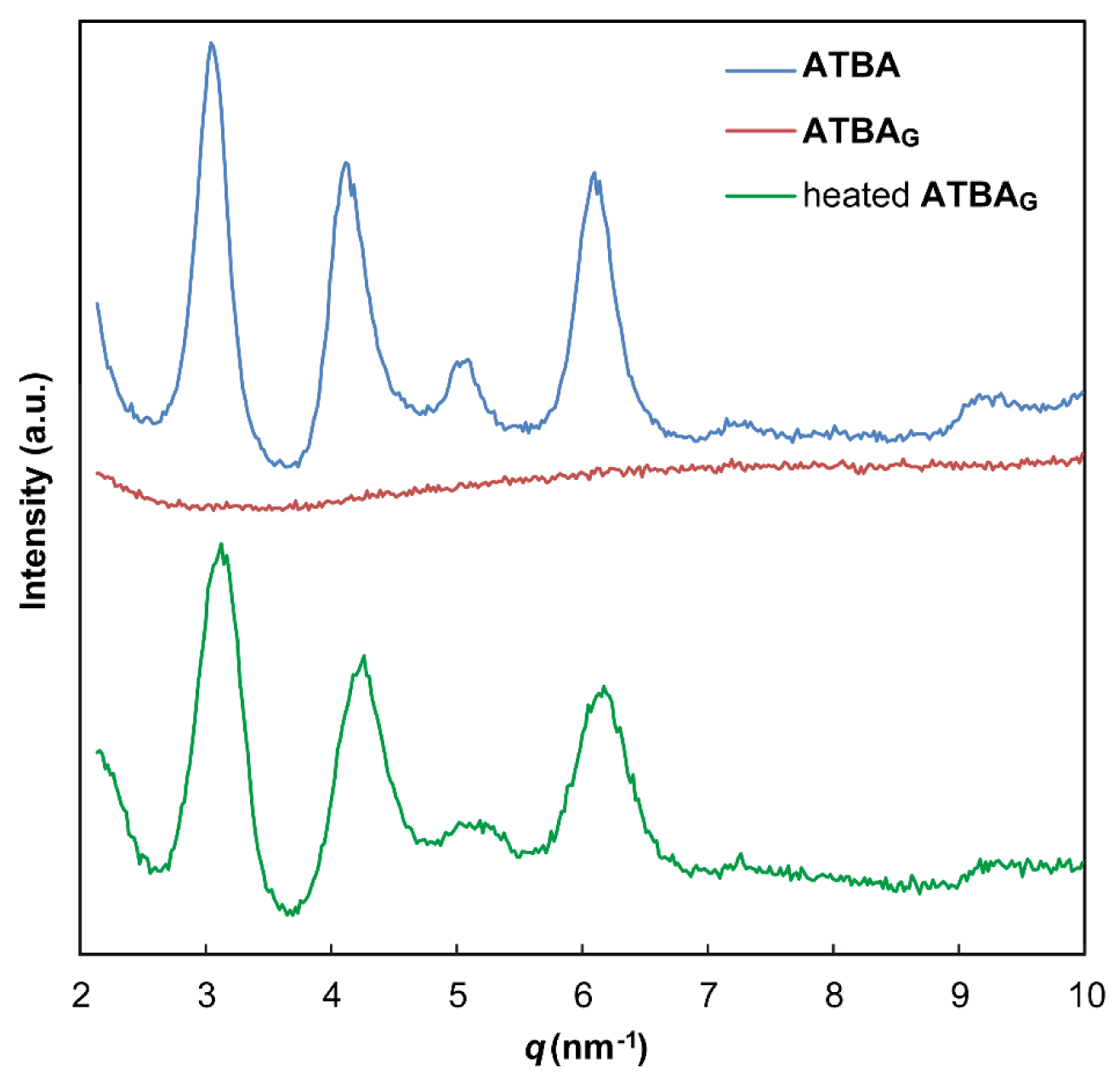

Supplement: S4 Fig — For sample preparation, an aqueous solution of ATBA (31 mg) was prepared in THF/water (3/1, 4 mL). ATBAG was formed by the slow evaporation of THF from the mixture at ambient temperature (ca. 20°C). Heated ATBAG was the white precipitate formed from ATBAG in the thermal cycle that involved heating it above 50°C and then cooling to room temperature. In the WAXS profile of the powder-state ATBA, the peaks attributed to the crystals in the powder appeared at 3.0, 4.1, 5.1, and 6.1 nm-1, whereas they disappeared in the profile of ATBAG. Thus, the structure of the aggregated material on a scale below 3 nm was likely to be different from that of the powder. In the WAXS profile of the heated ATBAG, the peaks appeared at 3.1, 4.3, 5.2, and 6.2 nm-1 and the position of these peaks were similar to that of powder-state ATBA. These results suggest that the structure of the white precipitate resembled the crystal structure in powder-state ATBA. (TIF) [file pone.0202816.s004.tif]

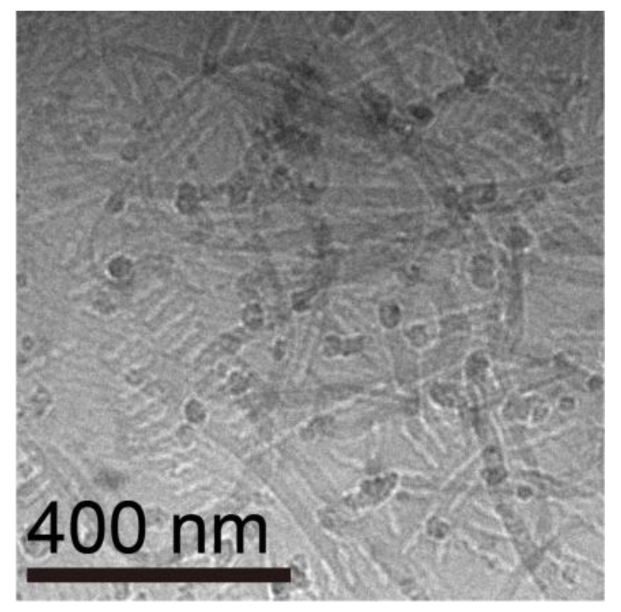

Supplement: S5 Fig — (TIF) [file pone.0202816.s005.tif]

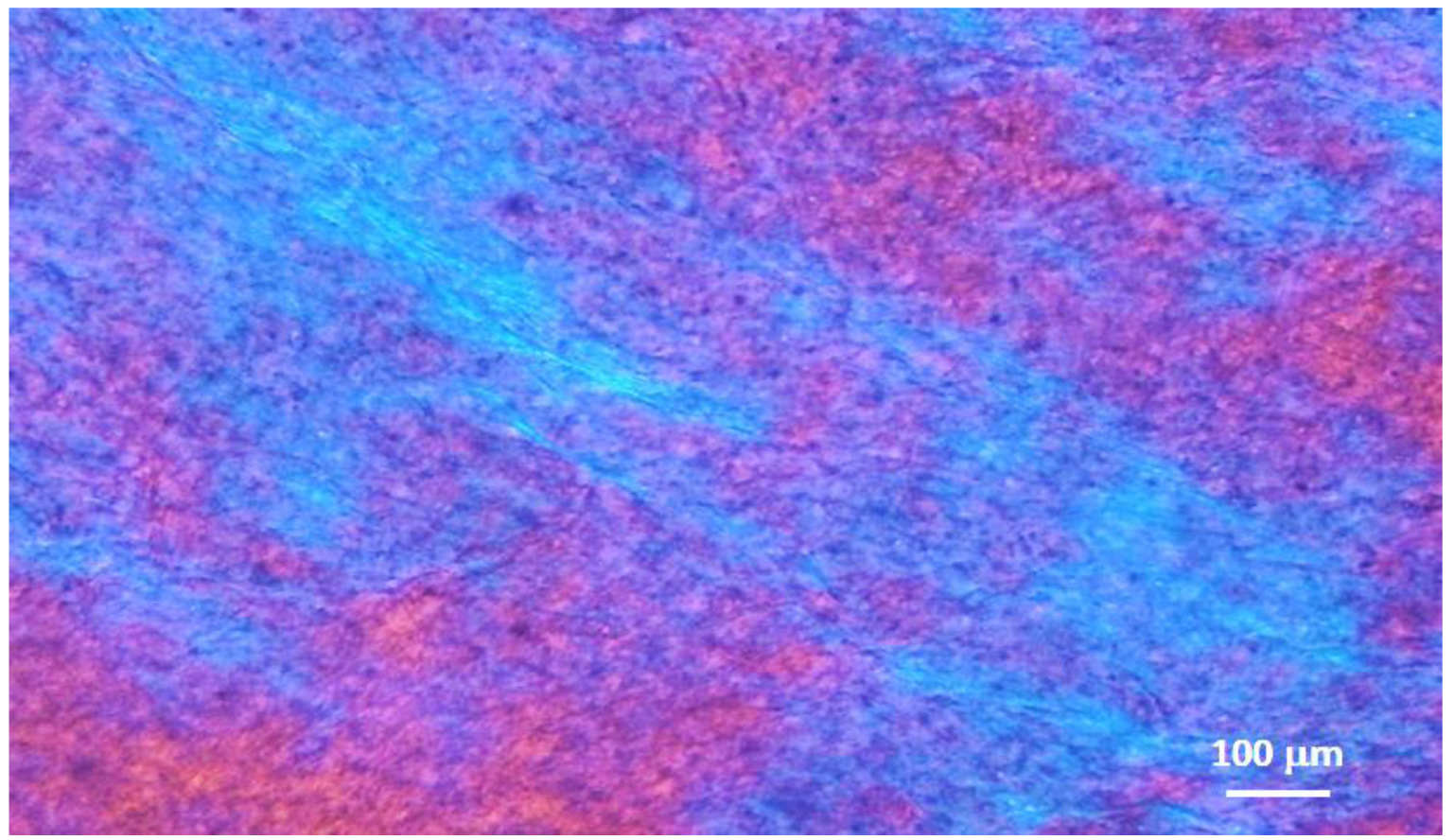

Supplement: S6 Fig — (TIF) [file pone.0202816.s006.tif]

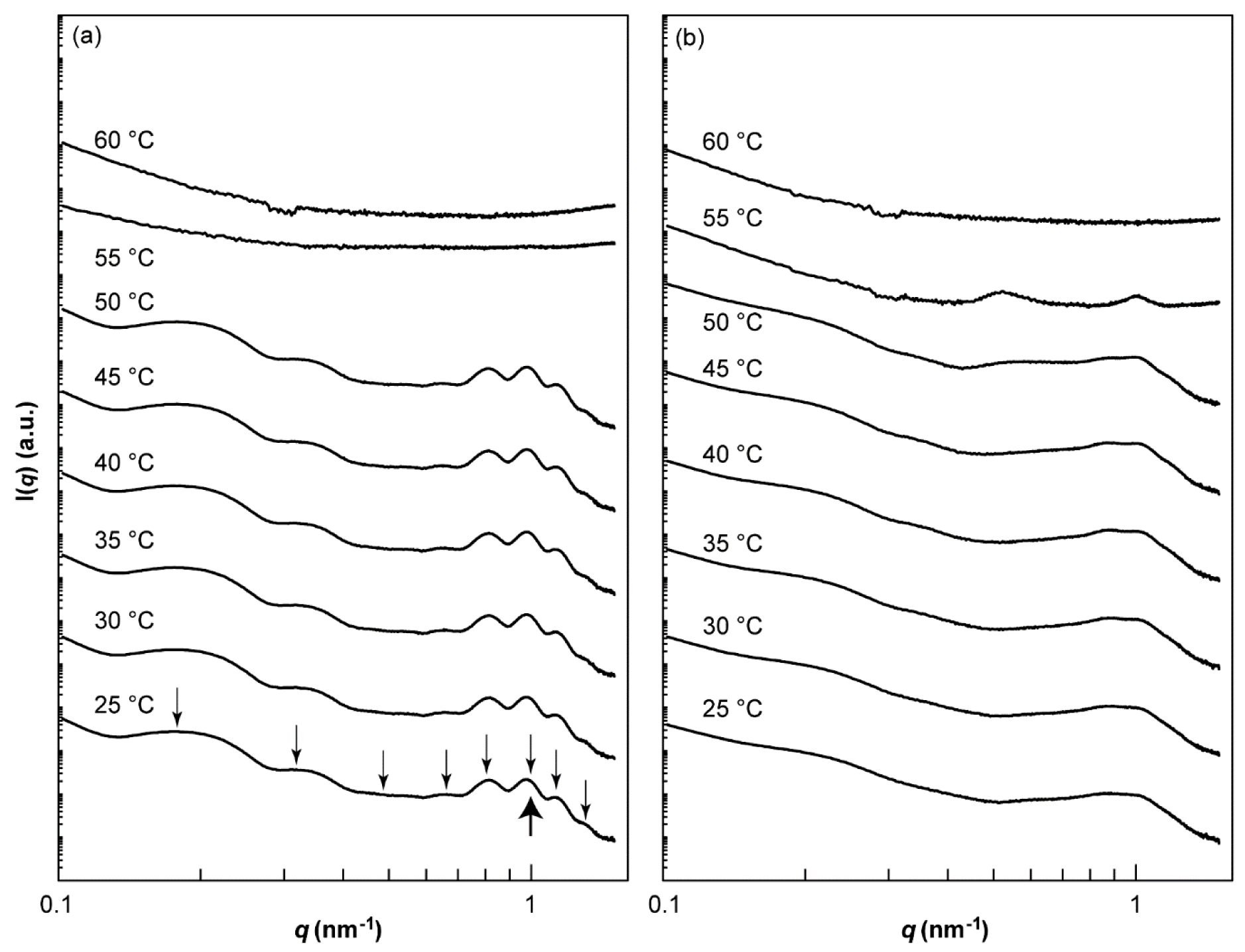

Supplement: S7 Fig — SAXS profiles of ATBAG with increasing temperature: ((a) first cycle, (b) second cycle). (TIF) [file pone.0202816.s007.tif]
